# Through the looking glass: empowering youth community advisory boards in Tanzania as a sustainable youth engagement model to inform policy and practice

**DOI:** 10.3389/fpubh.2024.1348242

**Published:** 2024-02-27

**Authors:** Dana Wai Shin Chow, Angelina Goi, Maeve F. Salm, Juma Kupewa, Getrud Mollel, Yassin Mninda, Judith Ambonisye, Alan Malongo, Eunice Ketang’enyi, Erica Sanga, Happyness Ngowi, Robert William, Emanueli Msuya, Blandina T. Mmbaga, Amos Mpili, Dorothy E. Dow

**Affiliations:** ^1^Duke-NUS Medical School, Singapore, Singapore; ^2^Kilimanjaro Christian Medical University College, Moshi, Tanzania; ^3^Duke Global Health Institute, Durham, NC, United States; ^4^Ifakara Health Institute Youth Community Advisory Board Liaison, Ifakara, Tanzania; ^5^Ifakara Health Institute, Ifakara, Tanzania; ^6^Mbeya Youth Community Advisory Board Liaison, Mbeya, Tanzania; ^7^Mbeya Zonal Referral Hospital, Mbeya, Tanzania; ^8^Mwanza Youth Community Advisory Board Liaison, Mwanza, Tanzania; ^9^Baylor College of Medicine Children’s Foundation Tanzania, Mwanza, Tanzania; ^10^Mwanza Research Centre, National Institute of Medical Research, Mwanza, Tanzania; ^11^Kilimanjaro Christian Medical Center-Duke Collaboration, Moshi, Tanzania; ^12^Moshi Youth Community Advisory Board Liaison, Moshi, Tanzania; ^13^IMPAACT Community Advisory Board Chair, Moshi, Tanzania; ^14^Kilimanjaro Clinical Research Institute, Moshi, Tanzania; ^15^Tanzania Ministry of Community Development, Gender, Women and Special Groups, Coordinator NAIA-AHW, Dodoma, Tanzania; ^16^Department of Pediatrics, Infectious Diseases, Duke University Medical Center, Durham, NC, United States

**Keywords:** youth engagement, youth community advisory board, youth empowerment, youth advocacy, Tanzania

## Abstract

More young people are living in the world than ever before, 90% of whom reside in low and middle income countries (LMICs). To address their needs, it is critical to have sustainable youth engagement when determining policy and to advance effective implementation of youth-focused interventions. Youth Community Advisory Boards (CABs) are a sustainable mechanism to achieve this goal. This paper describes engagement with youth CAB members across four locations in Tanzania. To set youth CAB meeting agendas and priorities, we asked youth CAB members to write (using free text) the top five challenges faced by young people in their communities (highest to lower priority). The Google Forms survey link was presented at the May 2023 youth CAB meeting and disseminated through WhatsApp. The survey was completed by smartphone, tablet, or paper provided to the youth liaison for data entry. Results were translated from Swahili to English and coded using excel. Findings were then presented back to the youth CABs at the September 2023 meeting. At that meeting, youth CAB members were then asked to write (free text) potential solutions to the most commonly described challenges. The surveys had response rates of 90% (84/93) for challenges and 78% (71/93) for solutions. The number one reported challenge was unemployment and financial instability (45%). Gender based violence (13%), sexual reproductive health issues (8%), and alcohol and drug use (8%) were in the top four both by priority and frequency of report. Other important challenges included physical and mental health, malnutrition, relationships, education, and societal and environmental norms, among others. Solutions included job creation, improved education, expanded legal systems, youth-friendly health care services, and increased social support through peer networks and community support. The National Accelerated Action and Investment Agenda for Adolescent Health and Wellbeing (NAIA-AHW) 2021/22-2024/25 includes most, but not all, of these top challenges and solutions. Ensuring young people have a seat at the policy table is critical to effective youth-empowerment in health and other related programs. Including a youth CAB member to represent this collective in youth-related government activities is a sustainable model to achieve this goal.

## 1 Introduction

There are more young people (10–24 years of age) today than at any point in history. One-fourth of the world’s 8 billion people are young people. Among them, 90% reside in low and middle income countries (LMICs) (1). Our collective future depends on the well-being of the upcoming generation. We must prioritize youth engagement, capacity building, and empowering young people to become catalysts for positive change within their communities.

Fostering youth engagement in research and health promotion improves health outcomes, self-perception, and mobilization of change (2). Furthermore, youth engagement in research improves young people’s agency over their health and reduces other youth-related challenges. This agency often develops into a sense of stewardship and responsibility toward the health of their peers and their community (3). Engaging young people in designing research also increases recruitment of young people from diverse populations, fosters youth-friendly messaging, and increases youth research participation (4, 5). Academic researchers who include youth also report an increased commitment, inspiration, and pride about their projects (6). Multiple benefits stem from prioritizing youth engagement in research, yet approaches to purposeful and sustainable youth engagement are lacking.

The three lens approach to youth engagement involves working for youth as beneficiaries, engaging with young people as partners, and supporting youth as leaders (7). All too often in policy or in research, young people are involved late in the decision making or design process, through a single focus group or stakeholder meeting, if they are engaged at all (8). This fragment of their time can be used to tick the box for youth engagement, but it rarely moves the needle and fails to critically inform youth-centered policy or intervention design and delivery. Genuine youth engagement requires “early and often” partnership with young people (9). Participatory research methods or incorporating young people into a research team with equal input on recruitment strategies or intervention delivery are productive strategies for research success (10). Engaging and training youth leaders as partners helps recognize their nuanced understanding of local contexts and challenges, while creating project ownership (11). Peer-led interventions carried out by trained youth leaders are also feasible, acceptable, and effective (12).

Establishing youth community advisory boards is an innovative strategy in extending young people’s engagement in research activities and community health improvement. Leveraging the success of the youth CAB in Moshi, Tanzania and partnering with the youth-informed and peer-delivered mental health intervention, Sauti ya Vijana (The Voice of Youth) in Tanzania (12, 13), we formed three additional youth CABs in each of the Tanzanian regions in which SYV is delivered. The mission of this youth CAB network follows the three lens approach (7): fostering greater youth agency in health through invited health talks for youth CAB members as beneficiaries, partnering with youth CAB members in youth-relevant research, and fostering youth leadership and advocacy through community outreach.

Through the partnership with this Tanzanian youth CAB network, our aim was to identify the most important health challenges faced by young people in Tanzania and summarize their recommended strategies and solutions. By comparing the health challenges named from youth CAB members with the National Accelerated Action and Investment Agenda for Adolescent Health and Wellbeing (NAIA-AHW) 2021/22-2024/25 (14), we hope to demonstrate the impact of partnering with young people to close the research to practice gap, empowering them to help shape the youth health agenda and share proposed interventions for young people.

## 2 Methods

### 2.1 Setting

Tanzania is a country located in East Africa. It includes over 60 million people of whom 20% are between the ages of 15–24 years and another 40% are 0–14 years (15). With 60% of the population under the age of 25, their health and engagement are paramount to the country’s future. The national language of Tanzania is Kiswahili, spoken by over 90% of people, but over 100 tribes contribute to the country’s culturally diverse environment.

### 2.2 Youth community advisory boards

**Figure 1:**
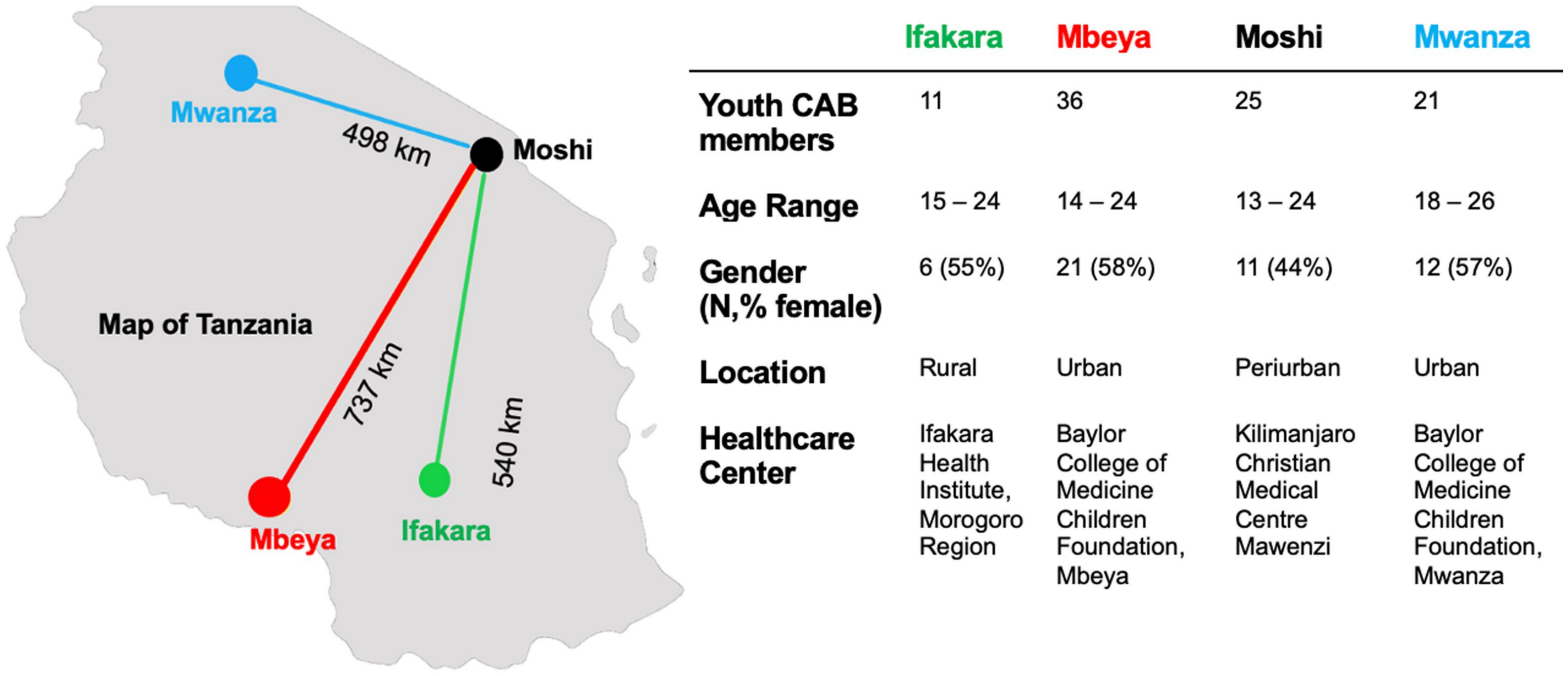
Location and composition of four distinct youth community advisory boards in Tanzania.

The network of youth CABs described in this manuscript operate in four regions of Tanzania: Morogoro, Mbeya, Moshi and Mwanza (Figure 1). Each site has an established partnership with a public, private, or public-private partnership healthcare institution. This facilitates a partnership whereby expert health providers are invited to speak to the youth CAB regarding important health topics, as suggested by CAB members themselves. Health institutions and researchers also reach out to the youth CAB members to advise on new study protocols and intervention design and delivery.

Respondents in this study are members of the youth CABs. Youth CABs in this network include young people between the ages of 13 to 26 years living with or affected by HIV (Figure 1). Members are selected in a site-specific manner, including recruitment from the HIV care and treatment clinics, invited by SYV stakeholders, selected through an interview process and/or recruited from schools. Youth CABs meet once a month and engage with local researchers, healthcare workers, and other policy makers to discuss research, important health topics, and shared lived experiences. Youth are compensated for their travel to meetings. The primary objective of the youth CABs is to incorporate the perspectives of young people in conversations about research and medical practices to ensure that policy decisions and research protocols better reflect the needs of the youth in their community. Subsequently, youth CAB members become stewards of health information, supporting the dissemination of research findings and bringing what they learned back to their communities through community events, targeted outreach projects, and radio talk shows.

**Figure 2:**
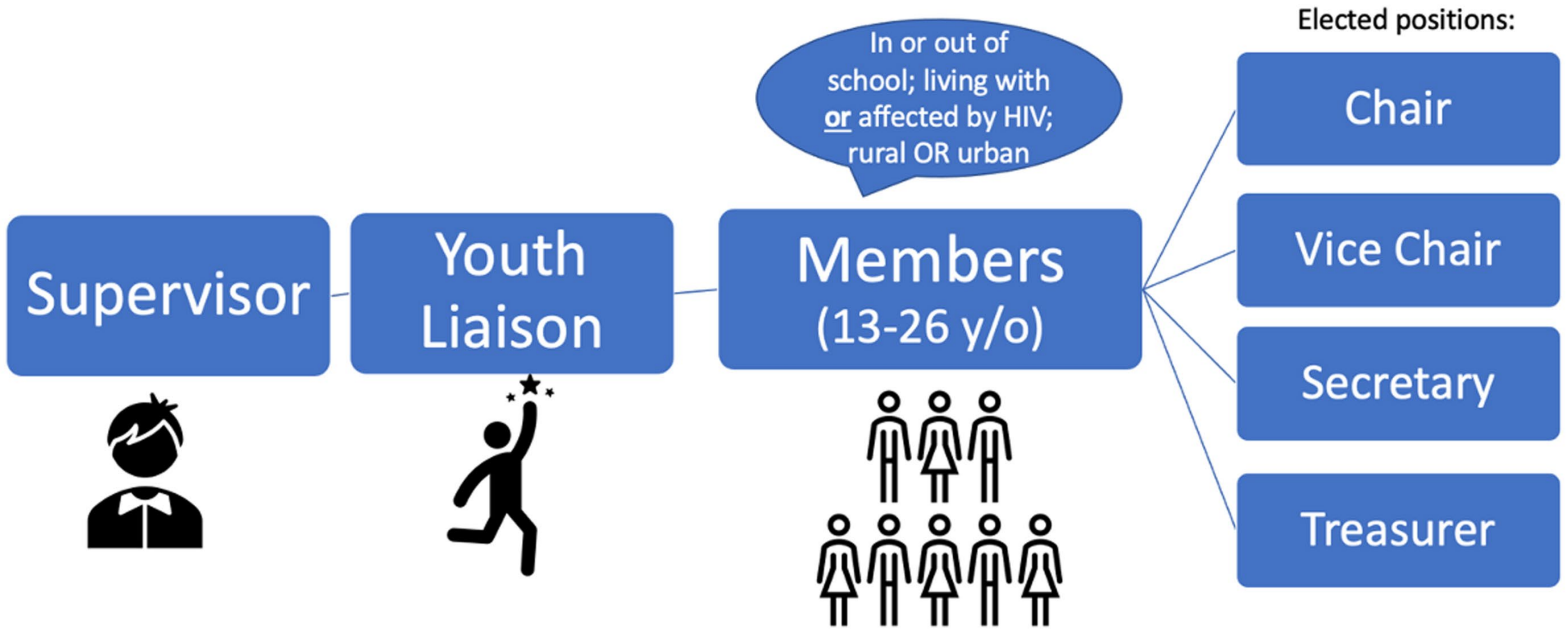
The organizational structure of youth CABs within this network.

Each youth CAB in this network follows a similar organizational structure (Figure 2). Site supervisors are local healthcare professionals and researchers who have volunteered time to support the liaison and youth CAB members in realizing their goals. Youth CAB members elect a chair, vice chair, secretary, and treasurer from their constituents to represent the voices of their membership within meetings and to help guide the youth CAB agenda. Each site also has a youth CAB liaison, a young adult champion that supports the elected youth CAB leadership in coordinating goal and agenda setting, budgeting, and other operational logistics (i.e., organizing the meeting location, transportation reimbursement, professional speakers at CAB, logistics for community outreach activities, etc.). Invited health topic experts are offered a small stipend for their time and travel to the meeting, teaching youth CAB members (beneficiaries) about desired health information. Researchers who utilize the youth CAB’s expertise (partners) pay a small fee that contributes towards sustaining youth CAB meetings, outreach activities, and travel expenses of youth representatives (leaders) in government meetings. All four site supervisors and liaisons report to the youth CAB network coordinator and the implementation team (located in Moshi, Tanzania).

### 2.3 Survey instruments and data collection

Two surveys were created in Kiswahili using Google Forms. The first survey was introduced at each youth CAB monthly meeting in May 2023. The link was sent to youth CAB members through WhatsApp, and each member was asked to submit, in order of priority, the top five challenges that young people face in their community (free text; Supplementary Figure S1A). The preliminary results were presented to each youth CAB at their monthly meeting in September 2023. A second survey, created using google forms, was again shared by WhatsApp and asked all youth CAB members if they agreed with the top five challenges and to describe possible solutions to the reported challenges (free text; Supplementary Figure S1B). Youth without access to a smartphone or internet shared devices or used a paper version. The youth CAB liaison entered any written responses into the electronic form. All members of the youth CAB network were eligible to participate. Respondents were asked to provide their gender and their site, but no other identifying information was collected.

### 2.4 Ethical considerations

Ethical approval for youth CAB formation and engagement was approved by the National Institute of Medical Research, protocol NIMR/HQ/R18c/Vol.I/2358, Bugando Medical Center/CUHAS under NIMR, Mbeya Medical Research and Ethics Committee (protocol SZEC-2439/R.A/V.1/128a), Kilimanjaro Christian Medical University College (proposal1311), Ifakara Health Institute Review Board (IHI/IRB/EXT/No: 33-2023), and Duke University (Pro00109309).

### 2.5 Analysis

Qualitative survey responses were reviewed in Kiswahili and translated to English using Google Translate. These translations were confirmed and corrected as needed by the youth CAB network coordinator (AG), who is fluent in both English and Swahili. Team members (DWSC and MFS) used applied thematic content analysis to inductively develop salient themes and subthemes for both surveys. After finalizing the “challenges” and “solutions” codebooks (Supplementary Tables S1, S2), two researchers (DWSC and MFS) independently coded and quantified survey results.

In the first “challenges” survey, each response corresponded to one challenge, which was coded as a single theme or sub-theme. This was in contrast with the second “solutions” survey where responses often included more than one solution to address the respective challenge, and thus were coded into as many themes as needed. The quantification of challenges themes was conducted first with responses listed as the top priority; then, with the most frequently mentioned challenges, even if the challenge was not the top priority. Discrepancies were resolved by additional team members (AG and DED) to reach a consensus.

Some entries appeared to be duplicated entries with the exact same free text. In such cases only one entry was included. Education as it related to health topics was coded in that health theme (for example education on contraception landed in “sexual and reproductive health (SRH);” health education landed in “general education”). HIV was mentioned in the context of SRH education, sexually transmitted infections, or stigma, so these responses were coded within the respective broader themes (e.g., SRH, Social Norms) (see Supplementary Tables S1, S2 for more examples).

## 3 Results

Youth CABs are composed of 93 members (12% from Ifakara; 39% from Mbeya; 27% from Moshi; 22% from Mwanza). Youth CABs are intended for youth 13–24 years. Slightly more females (54%) participate in youth CAB averaged across sites; however Moshi youth CAB has slightly more males (56%) (Figure 1).

### 3.1 Top challenges faced by Tanzanian youth

The first survey about the top challenges faced by young people was completed by a total of 84 participants, a response rate of 90% (*n* = 84/93) (Table 1). Approximately one-quarter of these responses were not differentiated by site due to an initial error in the survey that did not ask for this information initially but was quickly resolved. Slightly more males participated (53%).

**Table 1:** Survey results of identified challenges and solutions.

	Challenges survey			Solutions survey		
	Female (*n* = 40)	Male (*n* = 44)	Total respondents (*n* = 84)	Female (*n* = 35)	Male (*n* = 35)	Total respondents (*n* = 71)
Ifakara	2 (5%)	4 (9%)	6 (7%)	7 (20%)	6 (17%)	13 (18%)
Mbeya	14 (35%)	23 (52%)	37 (44%)	17 (49%)	14 (40%)	32 (45%)^*^
Moshi	5 (13%)	2 (5%)	7 (8%)	5 (14%)	7 (20%)	12 (17%)
Mwanza	10 (25%)	4 (9%)	14 (17%)	5 (17%)	8 (23%)	14 (20%)
Not recorded	9 (22%)	11 (25%)	20 (24%)	0 (0%)	0 (0%)	0 (0%)

^*^N = 1 youth CAB member from Mbeya listed both genders.

Tanzanian youth CAB members identified unemployment and financial concerns as the top priority or biggest challenge facing youth in their community. This was also the most frequently mentioned challenge among both male and female participants (Table 2). Gender Based Violence (GBV) was the second biggest challenge both by rank and frequency. Females mentioned this as a top priority more often than males (7 females vs. 4 males), but both males and females reported it as second biggest challenge by frequency (24 females vs. 26 males). Sexual reproductive health (SRH) was third highest top priority for females, while alcohol and drug use were the third biggest challenge for men. Other important themes included physical and mental health, education, malnutrition, and relationships (Table 2).

**Table 2:** Results of the top five challenges for Tanzanian youth by priority rank and frequency.

Priority #1		Gender			Overall frequency		Gender		
Rank	Themes	Female	Male	Total	Rank	Themes	Female	Male	Total
1	Unemployment and financial concerns	19	19	38	1	Unemployment and financial concerns	56	48	104
2	Gender based violence	7	4	11	2	Gender based violence	24	26	50
3	Sexual and reproductive health	4	3	7	3	Sexual and reproductive health	22	20	42
4	Alcohol and drug use	2	5	7	4	Alcohol and drug use	9	26	35
5	Physical health	4	3	7	8	Physical health	9	13	22
6	Mental health	2	3	5	10	Mental health	11	7	18
7	Food insecurity	0	4	4	12	Food insecurity	2	6	8
8	Romantic relationships, friendships and peer group influence	1	1	2	7	Romantic relationships, friendships, peer group influence	13	16	29
9	Religion and beliefs	0	1	1	15	Religion and beliefs	0	3	3
10	Education and skills	1	0	1	5	Education**	15	18	33
						Education and skills	11	12	23
						Business/entrepreneurship	4	6	10
11	Society and environment social norms	0	1	1	6	Society and environment	17	15	32
						Social norms	7	6	13
						Environment	4	6	10
						Lack of developmental support	6	3	9
					9	Family	13	7	20
					11	Youths’ self-perception	4	8	12
					14	Theft and violence	3	2	5
	Total	40	44	84		Total	190	234	424

^*^7 Respondent comments were uncoded due to 4 responded “none” and 3 were incoherent. ^**^Bolded text highlights the five most frequently mentioned topics as the first priority (on the left) and the five most frequently mentioned topics summed across all five priority responses (right).

#### 3.1.1 Unemployment and financial concerns

The theme of unemployment and financial concerns was overwhelmingly prioritized as the biggest challenge for young people in Tanzania. Responses highlighted a lack of employment and income-generating opportunities, particularly for young people. Examples in this category include “unemployment,” “many young people face the challenge of finding a job, especially after graduating from their studies,” and “employment for young people.” Respondents also wrote about financial barriers to education or self-employment as a challenge, mentioning, “higher education fees” and “capital to start various businesses,” reducing their opportunities to generate income or progress professionally. Respondents stressed that general economic instability was also a significant challenge for young people, quoting, a “lack of money,” “financial problem,” “economic problem,” and “poverty.”

#### 3.1.2 Gender based violence

Gender based violence (GBV) was the second most frequently described challenge faced by young people, and more commonly mentioned (7:4) as top challenge by females compared to males. Responses in this category ranged from acknowledging patriarchal norms to calling out violence taken against girls and young women. Youth wrote about “sexual harassment,” “GBV,” “FGM,” (female genital mutilation), “sexual violence,” “child marriage,” “rape,” and “lack of proper evidence on the roles of men and women in society.”

#### 3.1.3 Sexual and reproductive health

Respondents’ written responses emphasized that young people often lack knowledge and education about SRH, experience unintended pregnancies, and are affected by sexually transmitted infections (STIs). Written responses included “lack of contraceptive education,” “unexpected pregnancies,” “lack of education about reproductive health and family planning,” “menstrual problem,” “sex addiction,” “sexually transmitted diseases,” and “lack of awareness towards HIV and AIDs.” This category was mentioned as the top priority by seven respondents and 42 times overall, placing it as the third most significant challenge facing young people in Tanzania.

#### 3.1.4 Alcohol and drug use

In this category, respondents wrote about the consumption of substances (such as alcohol and other drugs), negative peer influences that encourage substance use, and learning how to support peers in discontinuing use. Written responses included “marijuana smoking,” “alcoholism,” “use of drugs and narcotics,” “smoking cigarettes,” “drug addiction,” “withdrawal,” “getting involved with illegal groups like smoking marijuana and alcohol,” and “how do we help young people who are addicted to drugs.” With seven respondents ranking this as top priority and 35 responses overall, this category was the fourth most frequently mentioned.

#### 3.1.5 Physical health

Respondents discussed general health, diseases, and a lack of health education as the main physical health challenges facing young people in Tanzania. Youth responses in this category were relatively vague, including comments such as, “health problems,” “outbreak of diseases,” “lack of health education,” “chronic diseases,” and “unhealthy living.”

#### 3.1.6 Mental health

Mental health was mentioned as a top priority by six youth (four male and two female) and overall mentioned 19 times fairly equally between males and females and across sites. Written responses in this category included “depression” and “mental health challenges.”

#### 3.1.7 Education

A need for increased education was another common response. Two distinct sub-themes made up this category: entrepreneurship education and skill-based and academic education. In the entrepreneurship category, respondents wrote about a desire for increased knowledge about self-employment. Responses included, “education on how to be self-employed” and “education for young people about entrepreneurship.” Skill-based and academic-related responses elucidated a lack of academic and professional development opportunities or support for young people, such as “lack of education,” “young people lack vocational and life skills,” and “young people drop out of school.” Though this was frequently mentioned (*n* = 33), it was the top priority for only one participant.

#### 3.1.8 Other notable challenges

Young people also highlighted that challenges in their built environment (“Society and Environment”) were difficult. Within this theme, respondents mentioned that a lack of developmental support, various social norms, and other environmental influences were significant concerns. Respectively, these concepts encompassed sentiments like “a lack of determining talents of youth at an early age,” “discrimination,” and “a difficult life situation” (Supplementary Table S1). Another prevalent challenge raised was related to navigating relationships and peer influences (“Romantic Relationships, Friendships, and Peer Group Influence”). Food insecurity, religion and beliefs, and theft and violence were also mentioned as challenges that young people face in Tanzania.

The majority of participants (97%) agreed with the top five challenges per survey results. The two responses that disagreed cited that there are “other priorities that affect society or the nation as a whole” and that “Young people are not fulfilling their dreams…they lack self-awareness and advice from close friends. You find he is getting into the wrong things.”

### 3.2 Solutions to the top challenges faced by Tanzanian youth

The second survey regarding solutions to the top challenges faced by youth had 71 responses (76%) (Table 1). Youth were asked to offer solutions to the challenges of unemployment, GBV, SRH, alcohol and drug use, and mental health (Supplementary Table S2). Responses across themes included a call for increased education about the respective challenge, such as entrepreneurship education (*n* = 22), sexual violence and sexual violence prevention education (*n* = 42), education on the impact of alcohol and drug use, education about various topics related SRH (i.e., contraceptives and STIs), and education on mental health challenges and coping strategies. Other solutions of note included a request for the provision of employment and loan opportunities for young people by the government or private sectors (*n* = 22), self-employment (*n* = 14). Youth-friendly service provision was also a top solution to encourage young people’s engagement with SRH services (*n* = 8), providing SRH services without discrimination based on age or partner status (*n* = 3), and improved access and referral to mental health services (*n* = 17). Improved legal frameworks that “establish strict laws for people who commit sexual violence” and easier ways to report “the government should have a number or social network where cases related GBV may be reported quickly.” Increased social and community support for survivors of GBV (*n* = 11) and of substance abuse (*n* = 2; Table 3).

**Table 3:** Proposed solutions for Tanzanian youth by priority rank and frequency.

Challenge	Suggested solutions	Sub themes	Female	Male	Total
Unemployment	Job creation and loan opportunity (both government and private sector)^a^	Create employment opportunities	15	7	22
		Provide accessible loans	14	6	22
	Improving education	Entrepreneurship	8	16	25
		Capital and loan	0	3	3
		Career development and jobs	5	7	12
	Youth ownership	In the youth’s hands (clarify)	3	6	9
		Self-employment	9	5	14
Gender based violence	Improving education	Sexual violence and prevention^b^	21	21	42
		Reporting and prosecuting	2	4	6
		Sex education	2	1	3
		Human rights and gender equity	3	2	5
		Discussing various beliefs	2	1	3
	Legal frameworks	Legal aid and avenues for reporting	2	7	9
		Enforcing laws^b^	5	3	8
	Support for survivors		5	5	11
	Religion		1	0	1
Alcohol and drugs	Improving education		21	21	42
	Legal frameworks	Law enforcement	6	9	15
	Employment	Employment to reduce idle time^b^	7	6	13
	Youth Ownership	Behavioral aspects	5	1	6
	Addressing mental health		1	2	3
	Support for substance users		1	1	2
SRH	Improving education	SRH, sex education, contraceptives and family planning, STIs	24	28	52
		Maternal health	5	2	7
		Providing education without discrimination (age, marriage, couples)	1	2	3
		Forming youth groups to teach peers	1	0	1
	Improving SRH services infrastructure	Encourage engagement with services	5	3	8
		Improving SRH service provision^b^	2	4	6
		Increasing manpower and training of medical professions	1	1	2
Mental health	Improving education	Mental health challenges	21	16	37
		Coping strategies and resources	6	8	14
	Improving mental health services infrastructure	Increase manpower and training of health professionals	2	3	5
		Provide early screening and diagnosis	6	3	10
		Improving access, treatment and referral patterns	7	10	17
	Healthy coping strategies	General social support	5	2	7
		Sports and exercise^b^	1	2	3
	Prevention strategies	Avoiding drugs and alcohol	0	1	1
		Reducing violence	1	0	1

^a^Some youth wrote govt, others private sector, others said both together; ^b^*n* = 1 youth listed both genders.

Across all themes, youth CAB members also recommended the formation of youth groups to provide and disseminate education, youth-driven solutions to the challenges (e.g., forming small entrepreneurship groups together, helping peers stop substance use), and other forms of community support for young people (e.g., community-wide acknowledgement of mental health challenges).

## 4 Discussion

Youth engagement is paramount to closing the research-to-practice gap for community health improvement. Involving youth voices in research ensures that youth-targeted interventions, services, and policies are acceptable, accessible, and align with their needs (16). Especially in Tanzania, where young people make up a pivotal portion of the population, youth engagement is a key feature of sustainable development and progress (17).

Tanzanian policy makers have created the NAIA-AHW 2021/22-2024/25 with the aim to develop and implement adolescent-specific interventions that target priority challenges faced by adolescents 10–19 years of age. The agenda is anchored on six pillars: (1) Preventing HIV, (2) preventing teenage pregnancy, (3) preventing sexual, physical, and emotional violence, (4) improving nutrition, (5) keeping boys and girls in school, and (6) developing soft skills for meaningful employment opportunities. Adolescent voices were incorporated into the development of the agenda, from the design phase and throughout implementation, by using focus groups, workshops, and online surveys.

Unemployment and financial concerns were overwhelmingly the top challenge identified by youth CAB members in our survey. It is similarly recognized as pillar six of the NAIA: to develop soft skills for youth to be better equipped to enter the workforce. Securing employment presents a formidable challenge for Tanzanian youth, stemming from a combination of limited opportunities in the labor force and disparities in education (18, 19). It is linked to many other challenges, including increased engagement in criminal activities, drug addiction, poor mental health, and increased suicidal rates (17, 20). As highlighted by the youth CAB, there is a disparity between skills acquired through formal education and the demands of the labor market, leading to a mismatch that impedes effective integration of young graduates into the workforce (17, 21, 22). NAIA’s sixth pillar aims to enhance employability and entrepreneurship by partnering with private sectors to seamlessly integrate life skills training into the education system. The youth CAB members similarly proposed creating opportunities for entrepreneurship training as a solution to unemployment. The NAIA also aims to strengthen vocational education and training and re-imagine a pre-existing soft-skills program called “Stadi za Kazi” meaning “work skills” to be more user friendly and integrated into primary and secondary school education (14). To compliment these NAIA interventions, youth CAB members also suggested that the government and private sector provide accessible loans.

GBV remains a pervasive issue in Tanzania, where 44% of Tanzanian women aged 15–49 have experienced physical violence, and less than 1% of those women sought GBV health care or legal services (23, 24). The young people in this survey highlighted this challenge and proposed solutions to address GBV that involve improving education and awareness, legal reform, and provision of accessible and youth-friendly support for survivors of sexual violence to create a safer and more inclusive environment. There have been growing efforts and awareness from the Tanzanian government to improve GBV support for women, such as the two current interventions featured in the NAIA’s third pillar to address physical, sexual and emotional violence. These two programs address the general concept of violence in society and incorporate GBV. One program through peer support groups that increase awareness and provide peer-to-peer support. The second program aims to strengthen protection systems to improve response and support services for GBV survivors. However, ensuring these interventions are well-implemented, accessible, and that young people are aware of them was a programmatic gap identified by the youth CAB members. Supporting this available programming, Youth CABs can be instrumental in bridging the gap by spreading awareness.

SRH was another significant challenge mentioned by majority of youth CAB respondents. Similarly, pillar two of the NAIA highlights the prevalence of teenage pregnancy, a topic mentioned by respondents within the scope of SRH. In Tanzania, almost half of all girls aged 19 are pregnant or have given birth to a child (24, 25). Overwhelmingly, the solutions offered by the youth CAB members to target SRH was improving education and access to youth-friendly reproductive services. The NAIA has suggested expanding access to comprehensive SRH information and delivering such topics through in-school and out-of-school curricula, with hopes to equip young people with the necessary tools to navigate complex social and cultural factors that contribute to early pregnancies. In addition, youth CAB respondents to this survey highlighted the need for increased awareness of and access to contraception. Family planning remains an evasive concept with a mere 8.6% of young people reporting contraceptive use and many facing barriers to SRH resources and treatment (26, 27). Only one-third of Tanzanian health facilities offer services for young people to obtain contraceptives, test for sexually transmitted infections, and receive the corresponding treatment (25). The NAIA aims to improve family planning skills and contraceptive use by promoting affordable sexual reproductive health services.

Although the majority of the prioritized areas in the NAIA corresponded with the issues mentioned by youth CAB respondents, there were a few discernible differences. A key challenge identified by the youth in this survey, but not as a pillar in the NAIA, was drug and alcohol abuse among Tanzanian youth. Though data is limited, estimated prevalence of substance use among adolescents aged 11–17 in Tanzania was 7%, and the 2012 drug control commission (DCC) highlighted that 96% of the total number of substance-dependent individuals were youth (28). The youth CAB respondents in this survey identified substance abuse as a challenge and attributed it to negative peer influence, unemployment, and consequently increased idle leisure time. The association between excess idle time and alcohol use is well documented, where young people who drop out of school or have difficulty finding employment are more susceptible to substance misuse (29). Moreover, youth CAB respondents highlighted the lack of public awareness on the risks of excessive drug and alcohol consumption and its effects, with several solutions aimed at improving education. Lastly, another contributing factor to youth substance use has been its accessibility, specifically easy access to alcohol, a lack of regulations or restrictions (30), and the frequent pull to entertainment establishments, which usually serve alcohol (31). Many youth CAB members advocated for the implementation of increased regulation on the sale and consumption of alcohol and other substances, demonstrating their potential to help address gaps in agenda-setting.

Mental health was another challenge highlighted by youth in this survey, but it was not an area of priority in the NAIA. Within sub-Saharan Africa, the prevalence of mental disorders among young people ranges between 13 and 20% (32). The youth CAB respondents highlighted education, improved access to mental health services, and treatment as vital to addressing the mental health crisis for young people. Integrating resources into school curriculum to improve mental health literacy have resulted in positive impacts on both students and teachers (32). There is a call to formalize mental health services in Tanzania, driving an ongoing but slow process of improving mental health services (33). Through task-sharing, several mental health interventions targeting Tanzanian youth have improved mental health outcomes. A school-based intervention in Northern Tanzania improved youth resiliency and prosocial behaviors (34). The use of trauma-focused CBT reduced adverse child mental health outcomes and post-traumatic stress disorder (35). An intervention with components of CBT, interpersonal psychotherapy, and motivational interviewing delivered by trained peer-leaders to groups of youth living with HIV has been piloted to improve mental health outcomes (12). Investing resources to improve mental health in adolescents can have lifelong health and economic benefits resulting in a high return on investment for the well-being of young people in Tanzania (36, 37).

Importantly, the NAIA’s first pillar focuses on HIV prevention, a topic that was mentioned relatively infrequently by the youth CAB members. A handful of responses (*N* = 5) directly mentioned HIV, highlighting its stigma and a lack of awareness around safe sexual practices. The Tanzania HIV Impact Survey 2016–2017 reports the incidence of HIV infection of youth 15–24 as 0.07% while the prevalence of HIV is about 2% among young people ages 15–19, increasing with age for both sexes (24). Notably, 50% of all new HIV infections in Tanzania are among the 15–29 year-old age group, the majority in young women (38). Our survey participants consisted of youth CAB members who are either living with or affected by HIV. Some young people did not specifically mention HIV as they had considered it a chronic disease or an STI, so these responses were captured within the broader themes of physical health and SRH. The relatively low prioritization of HIV-related challenges in the survey responses could also be attributed to participants’ focus on identifying the concerns of the wider community, rather than highlighting individual experiences. This phenomenon, however, underscores the broader issue where young people do not place HIV as a top priority due to the structural barriers discussed in this paper, such generating an income and sustaining their livelihoods.

The NAIA agenda is comprehensive and youth-informed, correlating with most of the challenges and solutions identified by the youth CAB respondents. Notably, the NAIA challenges and corresponding solutions are interconnected, such that if one area or “pillar” is improved, improvement will likely be noted in other areas. The six pillars are multifaceted, and none should be approached in isolation. However, it is important that young people as beneficiaries are aware of and able to help shape the interventions. Groups like youth CABs can help inform, partner, and disseminate critical information to ensure key programming reaches those who need it.

Our survey utilized partnership with youth CABs as a strategy to engage young people in research. It provided a dedicated platform for meaningful youth participation, amplifying their perspectives and centering their ideas to propel potential change and impact. The formation of youth advisory boards provides professional development and mentorship, fostering young leaders who advocate to effect change (39, 40). Facilitated through the Tanzanian Adolescent HIV Implementation Science Alliance (41), leaders of the youth CABs have the opportunity to engage with government officials in national youth-related agenda-setting and program implementation, such as the NAIA. Furthermore, many youth CAB respondents recommended the formation of youth groups as a place to educate young people about the respective topics or to serve as ambassadors of that health information. In other words, youth CAB members advocated for engaging youth CAB-like groups as a strategy to address the health challenges.

This study describes ways in which the youth CABs promote sustainable youth engagement to bridge the research and health services gap for young people. Youth CAB members provided information on key challenges faced by young people in their communities, serving as leaders by advocating for future youth beneficiaries. Youth CAB respondents proposed insightful and evidence-based solutions, demonstrating the importance of fostering partnerships between young people and policy makers to implement important interventions. Fostering youth engagement through mechanisms, such as youth CABs, will further develop young people into leaders, advising on research studies and interventions, disseminating health information, and advocating for community change. Young people are excited and capable of meaningful engagement in research (16). It is up to adults in positions of power to open the doors, add seats to the table, and invite young people’s perspectives, leveraging the power of 1.8 billion people for population health improvement (1).

### 4.1 Limitations

Data collection was limited by the inability to ensure unique responses to the survey as many youth CAB members did not have their own personal mobile devices and responded by paper form or shared a device, potentially resulting in duplicate responses. To address this, authors (DWSC and MFS) identified responses that matched exactly in terms of sequence and content and found one duplicate, which was removed. Another challenge was that the first survey was initially administered without recording the youth CAB locations, but this was rectified and subsequent responses were recorded, allowing the analysis of site specific data. The top five themes identified in the “solutions” survey were initially chosen and included mental health based on the preliminary findings from the “challenges” survey. After conducting multiple rounds of data analysis, the top five emergent challenges were slightly revised. Consequently, this led to “physical health” challenges as the fifth most important challenge, rather than “mental health” challenges. The finalized top five challenges were rectified following the dissemination of the second “solutions” survey, so solutions addressing “mental health” challenges were recorded rather than solutions to “physical health” issues. Nonetheless, capturing solutions to “mental health” challenges not found in the NAIA are beneficial to agenda-setting around young people’s health.

## 5 Conclusion

Youth CABs are an effective strategy for sustaining meaningful youth engagement and empowerment. Overall, the priority challenges highlighted by the youth CAB respondents were similar to that of the NAIA. A few key differences should be considered and addressed, such as substance use and mental health, in the next iteration of the adolescent agenda in Tanzania. Youth CABs as a collective and youth CAB leaders are insightful and powerful contributors to agenda-setting, serving as beneficiaries, partners, and leaders.

## Data availability statement

The raw data supporting the conclusions of this article will be made available by the authors, without undue reservation.

## Ethics statement

The studies involving humans were approved by National Institute of Medical Research, protocol NIMR/HQ/R18c/Vol.I/2358, Bugando Medical Center/CUHAS under NIMR, Mbeya Medical Research and Ethics Committee (protocol SZEC-2439/R.A/V.1/128a), Kilimanjaro Christian Medical University College (proposal1311), Ifakara Health Institute Review Board (IHI/IRB/EXT/No: 33-2023), and Duke University (Pro00109309). The studies were conducted in accordance with the local legislation and institutional requirements. Written informed consent for participation in this study was provided by the participants’ legal guardians/next of kin.

## Author contributions

DC: Formal analysis, Project administration, Writing – original draft, Writing – review & editing. AG: Formal analysis, Project administration, Writing – original draft, Writing – review & editing, Conceptualization, Data curation, Methodology. MS: Formal analysis, Project administration, Writing – original draft, Writing – review & editing, Supervision, Conceptualization. JK: Data curation, Supervision, Writing – review & editing. GM: Supervision, Writing – review & editing. YM: Data curation, Writing – review & editing, Supervision. JA: Supervision, Writing – review & editing. AMa: Data curation, Writing – review & editing, Conceptualization, Methodology, Supervision. EK: Supervision, Writing – review & editing. ES: Conceptualization, Supervision, Writing – review & editing, Methodology. HN: Writing – review & editing, Supervision. RW: Data curation, Writing – review & editing, Supervision. EM: Writing – review & editing, Supervision. BM: Writing – review & editing. AMp: Writing – review & editing. DD: Conceptualization, Formal analysis, Funding acquisition, Methodology, Project administration, Supervision, Writing – original draft, Writing – review & editing.
