# Supplementary material for: Through the looking glass: empowering youth community advisory boards in Tanzania as a sustainable youth engagement model to inform policy and practice
Source: Front Public Health. 2024 Feb 27;12:1348242. doi: 10.3389/fpubh.2024.1348242 (PMC10927807; doi:10.3389/fpubh.2024.1348242)
Supplement: Supplementary file 1 [file Table_1.DOCX]

**Supplemental Table 1. Codebook for the “Challenges” Survey**

| **Code** | **Sub-code** | **Definition** | **Examples** |
| --- | --- | --- | --- |
| Alcohol and Drug Use |  | Young people’s use of substances, such as alcohol, cigarettes, marijuana, and other drugs. Often, these responses spoke to abuse of substances or a desire to support peers in stopping misuse. | "Alcoholism and marijuana smoking," "How do we help young people who are addicted to drugs," "Drugs (withdrawal)," "Use of drugs and narcotics" |
| Education | --- | A lack of formal education and skills for young people. This theme does not include health education (included in physical health). |  |
|  | Education and Skills | A lack of academic or skill development for young people. This included young people dropping out of school, difficulties with academics, expressing a lack of employable skills among young people, and other examples. | "Education," "Lack of knowledge," "Young people lack vocational and life skills," "To be given more education in communication," "Lack of knowledge on our rights and how to take legal action in different encounters of life," "Young people drop out of school and become street people without a good direction in life," "Lack of science and technology education" |
|  | Entrepreneur-ship Education | A lack of education on self-employment, entrepreneurship, or business development for young people. | "Education on how to be self-employed as a young person," "Lack of entrepreneurship education" |
| Familial Challenges |  | Family conflict, a lack of caregiver support and guidance, and other family-based challenges. | "Not having a good foundation for the family," "Parents / guardians not supporting their children's talents," "Family problems," "Harassment by family," "Not getting good education for their foster parents, especially educationally as well as economically," "Lack of morals from bad upbringing,” "Not being listened to by parents/guardians" |
| Gender Based Violence (GBV) |  | Discrimination, violence, or other mistreatment of young women. Responses often highlighted sexual harassment or abuse as a result of the inequity in gender dynamics. | "Sexual violence," “Young women experience violence and they are abandoned with children by their partners," "Sexual bribery," "FGM" (female genital mutilation), “GBV,” "Not being given opportunities for girls in various opportunities," "Child marriage," "Lack of proper evidence on the roles of men and women in society," "Young people under the age of 17 are raped." |
| Malnutrition and Food Insecurity |  | Young people’s lack of access to food. | “Lack of food for young people,” “Malnutrition in young people,” "The life of young people is difficult to find food because many of us are not supported" |
| Mental Health |  | Adverse psychological outcomes, such as depression, among young people. | "Depression," "Young people face mental health challenges" |
| Physical Health |  | General health challenges, such as communicable and non-communicable diseases. A lack of general health education was included in this theme. | "Health problems," "Outbreak of disease in young people,” "Young people have a health challenge," "Lack of health education for young people," "Chronic diseases," "Lack of knowledge on the effects of unhealthy living, poor nutrition and risky behaviors" |
| Religion and Beliefs |  | Concerns about spirituality or comments on homosexuality. | "Not knowing God and having a relationship with God," "Many young people are attracted to homosexuality,” "Young people engage in same-gender sex" |
| Romantic Relationships, Friendships, and Peer Influence |  | Concerns about intimate relationships, adequate social networks, and negative peer influences. Negative peer influences often focused on unhealthy groups or activities. | "Relationship problems," "Romantic conflicts", "Young people are tempted to join bad groups like theft, rape and drunkenness," "Lack of good relationships in every area of their lives," "Peer pressure," "social problems" |
| Sexual and Reproductive Health (SRH) |  | Unintended pregnancies, STIs, and a lack of education about contraceptives or family planning. | "Menstrual problem," "Unintended childhood pregnancies," "Many young people are not educated about reproductive health," "Sexual diseases," "Lack of awareness towards HIV and AIDS," "Lack of contraceptive education for young women and men," "Lack of understanding about the effects of abortion" |
| Society and Environment | --- | Societal challenges that significantly impact young people. |  |
|  | Environment | Difficulties young people encounter in their surroundings, as well as concerns related to the health of the global environment. | "A difficult life that leads to joining destructive groups," "The increase of street children in our communities," "Difficult life situation," "Lack of transportation," "Lack of understanding to take care of the environment," "Environmental pollution in our communities" |
|  | Lack of Development-al Support | An articulated absence of guidance, support, and mentorship in moral, ethical, or individual development. | "lack of determining talents of youth at an early age," "Lack of mentorship in their lives in each of their areas," "Young people lack priority in community opportunities," "Lack of priorities for disadvantaged youth," "They are not involved in decision-making bodies," "Young people not being heard in their thoughts lead to a difficult life," |
|  | Social Norms | Common social expectations, beliefs, or practices that are challenging for young people. | "Discrimination," "Stigma," "Globalization," "Stigma in society," "Bullying of young people," "They are not trusted," "Stigmatization of young people," "anonymity," "Stigma for people living with HIV infection," "Humiliation" |
| Theft and Violence |  | Comments about violence toward young people and theft within communities. | "Cruelty," "Violence against young people," "A wave of theft in our communities" |
| Unemployment and Financial Concerns |  | A lack of employment or professional opportunities, particularly for young people, a lack of capital, education-related expenses, poverty, and other financial challenges. | "Unemployment," "Employment for youth," "The problem of employment after finishing studies," "Capital for Business," "Losing hope in life due to not having capital or jobs that can earn them income," "Poverty," "Financial problems," "Fee challenges and various requirements for students," "Capital for hiring to start various businesses," "Tuition fees in schools and colleges," "Lack of adequate opportunities for young people," "Lack of income |
| Youth Self-perception |  | Negative perspectives about young people and their behaviors, as perceived by young people. | "Not having the courage to do things that will help us succeed in life as a business," "Not having a vision and plans to achieve that vision," "Not identifying opportunities, using opportunities, solving challenges," "Lack of ability to determine opportunities and use the opportunities," "Youth cooperation," "They are not aware of the issues concerning the country," "Poverty due to the inactivity of the youth and love of games of chance more and laziness in the society," "Negative attitudes" |
