# Supplementary material for: Through the looking glass: empowering youth community advisory boards in Tanzania as a sustainable youth engagement model to inform policy and practice
Source: Front Public Health. 2024 Feb 27;12:1348242. doi: 10.3389/fpubh.2024.1348242 (PMC10927807; doi:10.3389/fpubh.2024.1348242)
Supplement: Supplementary file 2 [file Table_2.DOCX]

**Supplemental Table 2. Codebook for the “Solutions” Survey**

| **Challenge** | **Suggested Solutions** | **Sub-themes** | **Definition** | **Examples** |
| --- | --- | --- | --- | --- |
| Unemploy-ment | Job Creation and Loan Opportunity | Create employment opportunities | A recommendation to create or provide job or professional development opportunities for young people. This includes offering market insight, prioritizing young people in hiring, and other professional development activities | "The provision of small jobs that will enable young people to work so that even those who are not educated can make a living,” "Youth should be given opportunities," "Government and development stakeholders should strive to find local and foreign markets for young people,” "Giving youth priority on employment opportunities” |
|  |  | Provide accessible loans | A suggestion to provide capital or loans to young people. This recommendation arose most frequently in the context of providing accessible capital opportunities for youth entrepreneurship | "The government has to provide capital to young people so that they can be self-employed," "Financial institutions should help provide low-interest loans so that young people can support themselves" |
|  | Improving Education | Capital and loan education | Offering educational opportunities on how to access, obtain, and manage capital and loans. This response often arose in the context of youth entrepreneurship | "To provide capital education," "To be given loan knowledge," “To be given wealth management education" |
|  |  | Career development education | Providing education about career pathways, how to apply for and obtain employment, and other general information about entering the professional sphere | "Providing manual skills training even for those who have not studied to be able to use their skills,” "To get knowledge of what kind of job you will be interested to do," “Join various training groups” |
|  |  | Entrepreneur-ship education | Providing education about how to become an entrepreneur | "Entrepreneurship education,” "The government should provide entrepreneurship education to young people to enable young people to be self-sufficient in their communities," "Education of alternative ways to be self-employed" |
|  | Youth Ownership | Self-employment | A suggestion that young people seek opportunities to generate income through self-employment, creating options where they have onus over their ability to earn an income | "Young people to be able to engage in entrepreneurship to generate income,” "Young people should be able to engage in self-employment due to the opportunities around them in their communities" |
|  |  | Youth-driven solutions | Other actions that young people can take individually or together to generate income, educate themselves, or alter the landscape of youth unemployment | "Young people should formulate groups to find job and market for their small business to eliminate poverty," "Creating groups and helping each other in perspective and also helping each other in practice," "Young people should organize entrepreneurial groups" |
| Gender Based Violence | Improving Education | Discussing various beliefs | Providing education and spaces to have open dialogue about perspectives on gender, culture, and practices that impact the ways genders move through society and GBV | "The government should create groups to be able to help and eradicate sexual violence through practices with false beliefs," "Education about African systems and cultures that oppress gender," "Abandoning customs and misconceptions" |
|  |  | Human rights and gender equity | A recommendation to educate individuals about human rights and gender equity, including individuals’ right to bodily autonomy | "Society and institutions should provide gender education, especially in schools," "Education should be given to young women and men as soon as they reach the age of puberty to know what is their right and what is not their right," "Parents are aware that all genders have equal rights," "Self-awareness and adherence to human rights," "Educating young people about sexual rights for all" |
|  |  | Reporting and prosecuting | Providing education about how individuals can report instances of gender-based violence and pursue accountability measures for those experiences if desired | "Communities should be educated on the laws in place regarding sexual violence," "Education should be given to know the places to prosecute when there is a challenge" |
|  |  | Sex education | A recommendation to provide information about sexual intercourse to reduce instances of gender-based violence (e.g. sexual violence, rape) | "Providing sex education for youth and society in general" |
|  |  | Sexual violence and prevention | Providing education on what sexual and gender-based violence are as well as how to prevent instances of violence from occurring | "To provide education on sexual violence,” "Providing education to young people about how to fight sexual violence and its effects to help them deal with it," "Education on sexual violence should be given to rural communities," "It is necessary to learn about sexual violence in schools, in the villages," "Society needs education on sexual violence and how to combat these challenges" |
|  | Legal Frameworks | Avenues for reporting | A recommendation to improve existing mechanisms and implement additional ways to report instances of GBV | "There are desks to defend gender rights so that people can come to report violence they encounter in the community," "The government should have a number or a social network where cases related to GBV may be reported quickly" |
|  |  | Upholding laws | A suggestion to improve the adherence to and enforcement of laws related to GBV. This also includes a call for increased laws related to violating gender-based rights and greater legal accountability for these violations | "Establish strict laws for people who commit sexual violence to prevent the increase of sexual violence," "The existence of civil organizations that bear the responsibility of receiving cases," "Abiding by the laws laid down without regard to corruption" |
|  | Religion |  | Examining the ways that religion intersects with GBV | "Sexual violence in our society is due to a lack of fear of God, but also superstitious beliefs, we must provide a place for the end of this practice" |
|  | Support for Survivors | --- | A call for increased and improved support for survivors of GBV |  |
|  |  | Social support | A recommendation to improve social mechanisms of support for survivors of GBV. This includes mostly informal mechanisms for support, like altering social norms to encompass intervention when GBV is observed | "The government should create youth to mobilize the community," "Everyone to be the ambassador and protector of each other," "Everyone to be an ambassador and a watchdog at any of the events and to report on the relevant areas" |
|  |  | Structural support | A request for increased institutional mechanisms of support for survivors of GBV, such as provision of medical services | "People who are subjected to acts of violence can be helped with early treatment to avoid psychological problems," "The government should improve medical services for people who have been subjected to sexual violence," "A better mechanism for providing information to those who are subjected to sexual violence such as simple online desks such as a phone service or texting without a charge," "The existence and availability of gender desks for those who were subjected to such violence" |
| Alcohol and Drug Use | Addressing Mental Health |  | A recommendation to address adverse mental health outcomes of young people to prevent use and abuse of substances | "The government should limit stress to society to reduce the use of alcohol," "Many young people experience depression and see that drinking alcohol is what makes them feel better" |
|  | Employment |  | A suggestion to increase employment among young people to reduce their substance use | "There should be a lot of work for young people so that they are busy with more responsibilities," "If youth are employed, they will spend a lot of time at work and reduce the time to drink" |
|  | Improving Education |  | Providing information about the impact of substance use on individuals, ways to reduce substance use, and how to support peers in reducing their substance use | "Provide education on the effects of drug use," "Young people should be given education to protect themselves against drugs and alcohol use in general," "The health sector should create youth groups that will help provide education to the community about the abuse of alcohol and drugs" |
|  | Law Enforcement |  | The creation or enforcement of regulations on substance sale, purchasing, or consumption | "The government should enact laws and bans that will reduce the use of alcohol and drugs," "The government should prevent the production and processing of illegal alcohol and drugs," "The government should put in place laws on the use of alcohol, such as a time limit for people to use those intoxicants," "Banning and taking disciplinary action against producers and consumers," "Creating a law that will help young people" |
|  | Support for Substance Users |  | A call for increased support for individuals who use or abuse substances to help them reduce their use | "Counselling and finding out what is the root cause of the problem," Sending them to rehab," "Educational support for young victims" |
|  | Youth Behavioral Aspects |  | Recommended behavior changes among young people to facilitate a reduction in substance use | "Stay away from groups with dangerous behavior," "The government should find a way to make young people focus on work" |
| SRH | Improving Education | Provide education without discrimination | A call for access to sexual and reproductive health education without restriction based on partner status, age, or other socially-driven expectations | "Providing education to all ages without any discrimination," "Health Organizations should provide education without discrimination of different ages because maternal health education is not only for married people," "The society should provide education not only to couples but also to young people studying in primary and secondary schools" |
|  |  | SRH, sex education, contraception, family planning, STIs | A recommendation for increased provision of and access to sexual and reproductive health education for young people, including topics such as information on safe sexual intercourse practices, contraceptives, sexually transmitted infections, and other information related to family planning and SRH | "Getting education about birth control," "Being able to provide education about family planning in schools," "Youth should be given reproductive health education," "Providing education in the hospital as well as in the community," "Education should be provided on how to protect yourself from reproductive health problems, such as sexually transmitted diseases, cancer and fungal diseases that cause attacks on reproductive health," "The community should form youth groups about reproductive health and go to teach their peers," "Providing family planning education for young people," "Maternal education should be given to young people," "Parents should provide education to their children in the home without fear and transparency," "Young people can be given comprehensive sexual health education" |
|  | Improving SRH Services | Encourage engagement with services | A suggestion that young people use sexual and reproductive health services offered in order to obtain resources they need | "People are encouraged to go for reproductive health screening," "Have time to go to check reproductive health regularly," "Go to the hospital for further advice," "Young people can visit health centers on a regular basis" |
|  |  | Improving SRH service provision | A call to increase access to sexual and reproductive health services by providing services in more facilities and by providing a greater breath of services | "They should be given condoms," "There should be access to medical services on reproductive health" |
|  |  | Increasing manpower and training of medical professions | A recommendation to increase the number of medical professionals who provide SRH services in order to increase access to services | "The government must be able to bring in enough doctors and educators to be able to improve the problems of reproductive diseases" |
| Mental Health | Healthy Coping Strategies | General social support | A suggestion that young people seek out support from their friends, peers, and others in their social networks in order to address their mental health challenges | "Share the challenges you are going through with others," "Positive groups to build young people," "A young person should get advice from close friends," "Collaborate with people and talk to someone you trust when you have a challenge" |
|  |  | Sports and exercise | A suggestion that young people seek out sports or other forms of exercise as a way to cope with their mental health challenges | "The presence of groups that encourage exercise to practice makes one's mind grow," "Sports and other ways to keep young people occupied" |
|  | Improving Mental Health Services | Improving access, treatment and referral patterns | A recommendation to increase access to mental health services as well as improve mental health services | "Treatment should be provided to the community and the government should give priority to departments related to mental health," "Psychosocial services should be provided in youth, family and school groups," "Availability of mental health treatment for young people," "Connect [young people] with mental health professionals and psychologists," "The government has reduced the cost of mental health," "The community helps people with mental health to get them to health facilities" |
|  |  | Increase manpower and training of health professionals | A call to increase the number of medical professionals who can address adverse mental health outcomes in order to improve access to mental health services | "An increase in the number of service providers in the Department of Mental Health so that they can reach everywhere at the right time," "Nurses should be empowered and there should be more for the community to make it easier to deliver education to the community," "Engage with psychologists to mentor young people," "The government should priority mental health providers (doctors)" |
|  |  | Provide early screening and diagnosis | A call to provide preventative screenings to identify existing mental health challenges and improve the time between symptom onset, referral, and receipt of treatment | "Research and education in the community should be given on identifying people with mental health early so that they can help them quickly," "People with symptoms of mental health disorders should be diagnosed early so that they can receive treatment that will be able to help them before the problem becomes serious" |
|  | Improving Education | Coping strategies and resources | A recommendation to provide education about strategies and resources to cope with mental health challenges for young people | "Education should be given to the community so that they can deal with the problem of mental health," "Teaching about how to cope with stress," "Young people should be given education on how to deal with mental health challenges" |
|  |  | Mental health challenges | A recommendation to provide more education about the existence and symptoms of adverse mental health outcomes for young people and their community | "Young people should be given mental health education," "Mental health education should be provided to the community as the problem is chronic and growing rapidly," "The government should be given education about mental health and examine mental health," "Providing more information on the causes and effects of mental health," "The community should accept that there is a mental health problem so that if they are educated, they can receive and accept treatment" |
|  | Prevention Strategies | Avoiding drugs and alcohol | A suggestion that young people avoid substance use as a mechanism to reduce mental health challenges | "Young people should not use drugs and alcohol" |
|  |  | Reducing violence | A suggestion for reduced violence against young people as a mechanism to reduce mental health challenges | "Investigations should be done early for those who are treated badly or are in a dangerous situation" |
