# Supplementary figures and images for: Through the looking glass: empowering youth community advisory boards in Tanzania as a sustainable youth engagement model to inform policy and practice
Source: Front Public Health. 2024 Feb 27;12:1348242. doi: 10.3389/fpubh.2024.1348242 (PMC10927807; doi:10.3389/fpubh.2024.1348242)

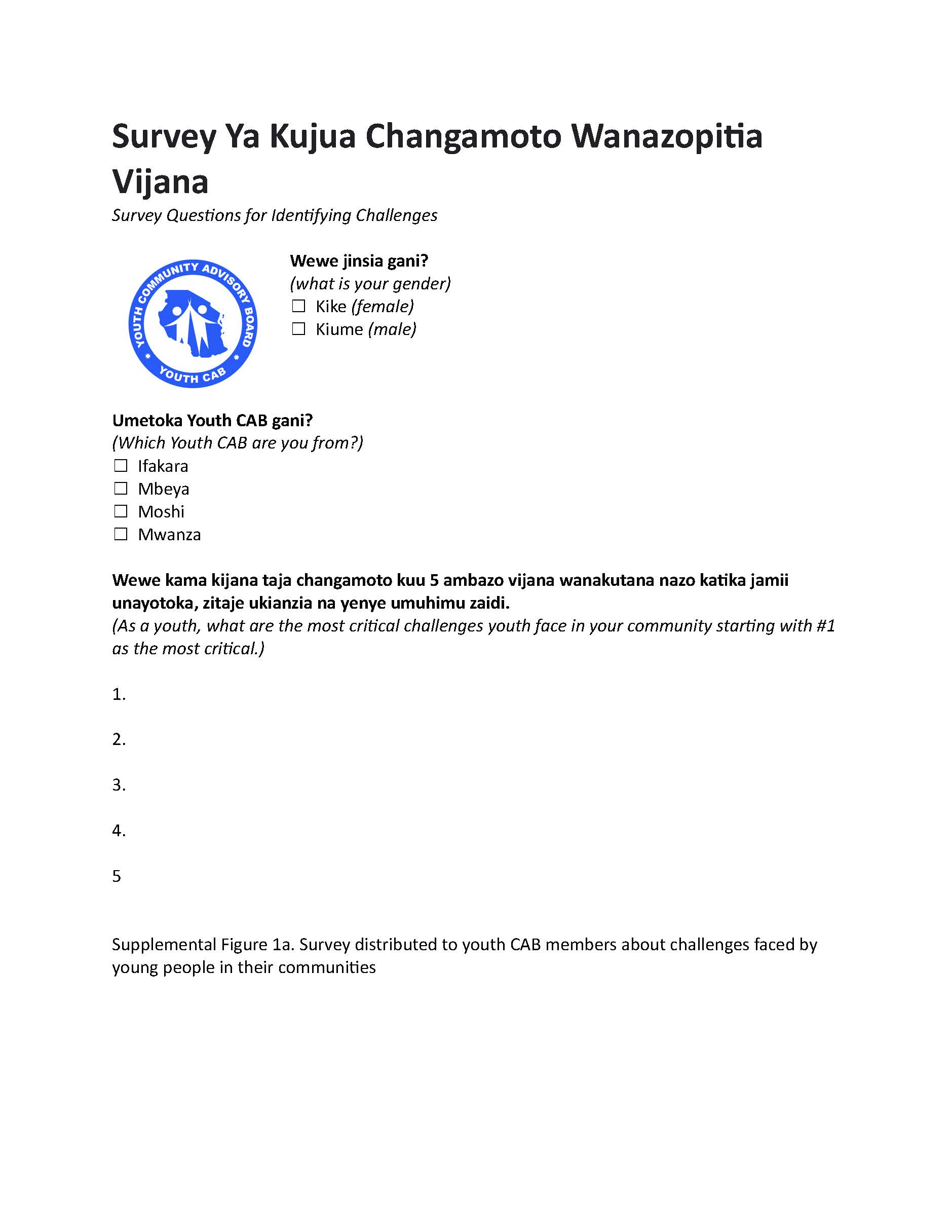

Supplement: Supplementary file 3 [file Image_1.JPEG]

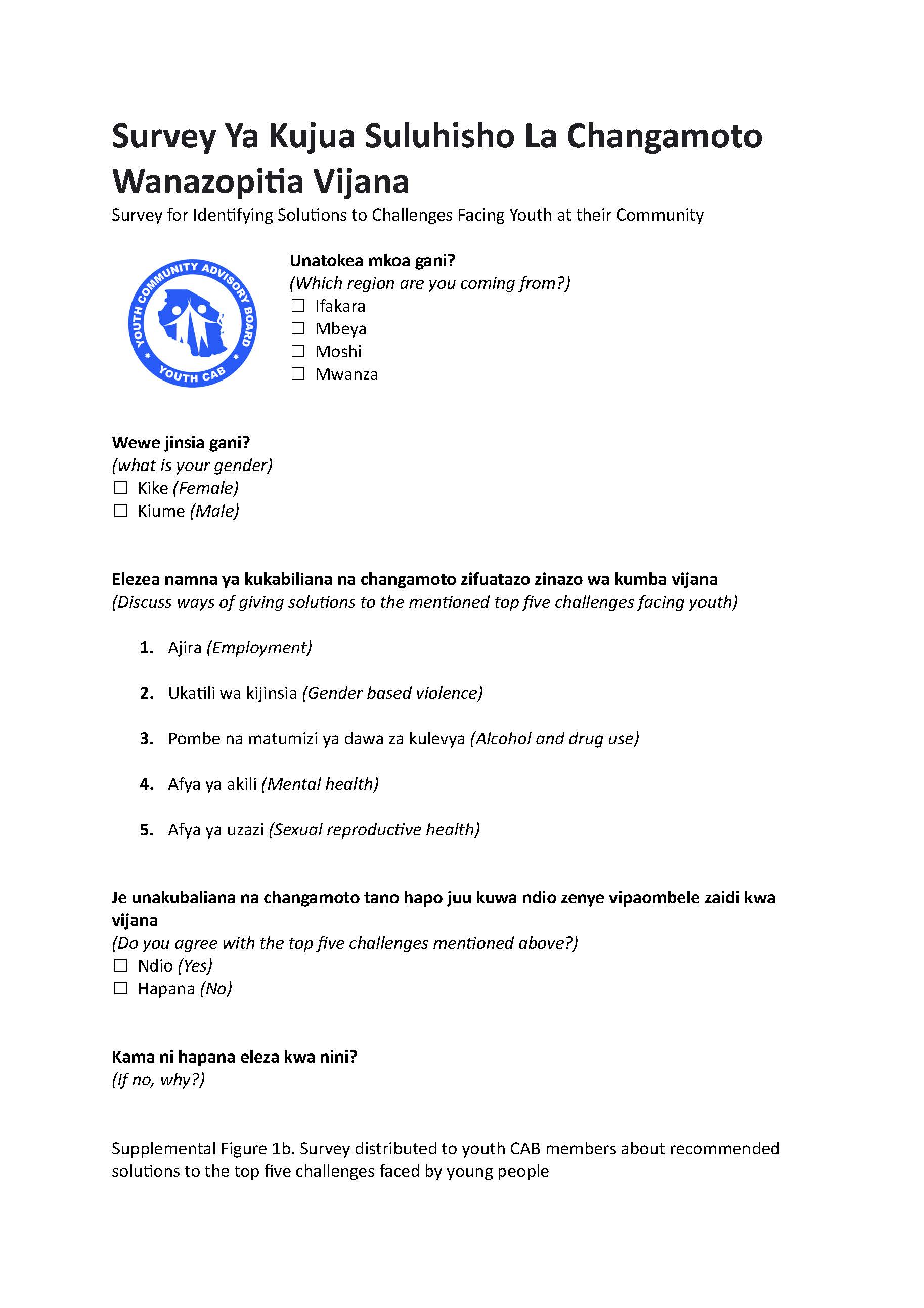

Supplement: Supplementary file 4 [file Image_2.JPEG]
